# Computational Modeling of the Electrochemical System of Lipase Activity Detection

**DOI:** 10.3390/s8063873

**Published:** 2008-06-09

**Authors:** Mantas Puida, Feliksas Ivanauskas, Ilja Ignatjev, Gintaras Valinčius, Valdemaras Razumas

**Affiliations:** 1 Faculty of Mathematics and Informatics, Vilnius University, Naugarduko 24, LT-03225 Vilnius, Lithuania; 2 Institute of Biochemistry, Mokslininkų 12, LT-08662 Vilnius, Lithuania

**Keywords:** Amperometric biosensor, lipase activity, ferrocene-based substrate, modeling, simulation

## Abstract

This paper presents computational modeling of response kinetics of bioelectroanalytical system based on solid supported lipase substrate and lipase interaction. The model assumes that lipase substrate is formed by dripping and drying a small amount of the ethanol solution of 9-(5′-ferrocenylpentanoyloxy)nonyl disulfide (FPONDS) and that lipase is capable of cleaving FPONDS ester bonds via hydrolysis mechanism. Two mathematical models have been developed and evaluated trough computational simulation series by comparing them to experimental data. The results of simulation demonstrate that a good fitting might be obtained only taking into account non-linear substrate wash off process.

## 1. Introduction

Lipolytic enzymes are one of the most important components of the biochemical processes. At the same time, triacylglycerol acylhydrolases (EC 3.1.1.3) that hydrolyze triacylglycerols at the oil/water interface have wide applications as detergent additives, digestive aids, as well as in the paper and food industries [1-3]. Unlike other bond-cleaving enzymes, e.g., proteases, hydrolysis by lipases is carried out in heterogeneous multiphase systems. In many cases, the environment of the enzyme at the substrate/liquid interface plays an important role for the overall enzymatic activity of these proteins [1-4]. Thus, the ability to monitor enzymatic activity of lipases under these conditions is of paramount importance.

Recently, a novel electrochemical technique for the assay of lipase activity has been described [5]. The method utilizes a solid supported lipase substrate, which is formed by dripping and drying a small amount of an ethanol solution of 9-(5′-ferrocenylpentanoyloxy)nonyl disulfide (FPONDS; [Fc-(CH_2_)_4_COO(CH_2_)_9_S-]_2_, where Fc is the ferrocene) on the gold electrode surface modified by a hexanethiol self-assembled monolayer. The redox-active ferrocene group of FPONDS generates the amperometric signal, the intensity of which is proportional to the number of FPONDS molecules at the interface. Electrochemical and surface-enhanced infrared absorption spectroscopic data, as well as control experiments with an engineered, deactivated mutant enzyme, have demonstrated that the wild-type lipase from *Thermomyces lanuginosus* (TLL) is capable of cleaving the ester bonds of FPONDS molecules via an enzymatic hydrolysis mechanism, which includes the adsorption of the lipase onto the substrate surface. The interfacial enzymatic process liberates ferrocene groups from the electrode surface triggering a decay of the electrochemical signal. The rate of the electrochemical signal decrease is proportional to the lipase activity.

However, in exclusively experimental work [5], no kinetic model has been proposed to account for the features of amperometric biosensor response, namely, current decay vs. time upon enzyme action. This paper is intended to fill this gap.

## 2. Kinetic model

**Figure 1.**
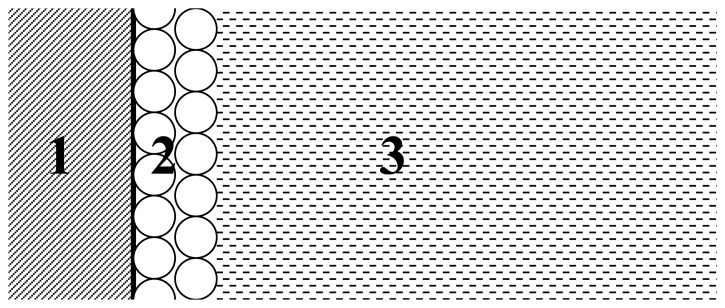
Cross-section scheme of the model used in the present study: **1.** gold electrode, **2.** FPONDS substrate layer(s), **3.** lipase solution.

This paper analyzes bioelectroanalytical system that is significantly different from recently discussed amperometric system of lipase activity determination [6], where enzyme acts on the surface of substrate-bearing micelles spread in the solution. Currently modeled system is schematically presented in Fig. 1.

The processes that occur at the interface of zones 2 and 3 could be described in the following schematic form which is most commonly used for the description of lipase interfacial activation [7]:

(1) $${\text{E}}_{\underset{k_{D}}{⟶}}^{\overset{k_{p}}{⟵}}\;\text{E}*$$

(2) $$\text{E}*+{\text{U}}_{\underset{k_{-1}}{⟶}}^{\overset{k_{1}}{⟵}}\;\text{E}*\text{U}\overset{k_{2}}{⟶}\;\text{E}*+\text{P},$$

where E is the enzyme in solution, E * is the enzyme attached to the surface of substrate (at the interface of zones 2 and 3 in Fig. 1), U is the ferrocene-based substrate FPONDS substrate on the gold electrode surface, E*U is the enzyme-substrate complex, and P represents the reaction product. The change of U concentration as a function time is the object of our computational simulations as it is directly proportional to experimentally registered electrode signal (see, for instance, Fig. 1 in Ref. 5).

It is assumed that lipase solution is distributed evenly and its diffusion could be not taken into account. It is also assumed that the redox-active reaction product (ferrocene-based) leaves sensor surface quite fast and its diffusion could be estimated as instantaneous. The system under discussion can be described by classical mathematical model of reaction kinetics:

(3) $$\{\begin{array}{l} \frac{dE^{*}}{dt}=k_{-1}\;E^{*}U+k_{2}\;E^{*}U-k_{1}\;E^{*}\times U+k_{p}E-k_{D}\;E^{*}\\ \frac{dE^{*}U}{dt}=k_{1}\;E^{*}\times U-k_{-1}\;E^{*}U-k_{2}\;E^{*}U\\ \frac{dU}{dt}=k_{-1}\;E^{*}U-k_{1}\;E^{*}\times U\\ \frac{dP}{dt}=k_{2}\;E^{*}U\\ \frac{dE}{dt}=\left(\frac{S}{V}\right)k_{D}\;E^{*}-\left(\frac{S}{V}\right)k_{p}\;E \end{array}$$

where symbols in italics *E*, E*, E*U, *P* and *U* represent concentrations; *S* is the interfacial area of electrode; *V* is the total volume of solution; *k_p_* is the rate constant of enzyme adsorption at the electrode surface, *k_D_* is the enzyme desorption rate constant, *k*_1_ is the rate constant of enzyme-substrate complex (E*U) formation, *k*_-1_ is the rate constant of E*U dissociation, *k*_2_ is the catalytic rate constant of enzymatic reaction, and *t* is time.

**Figure 2.**
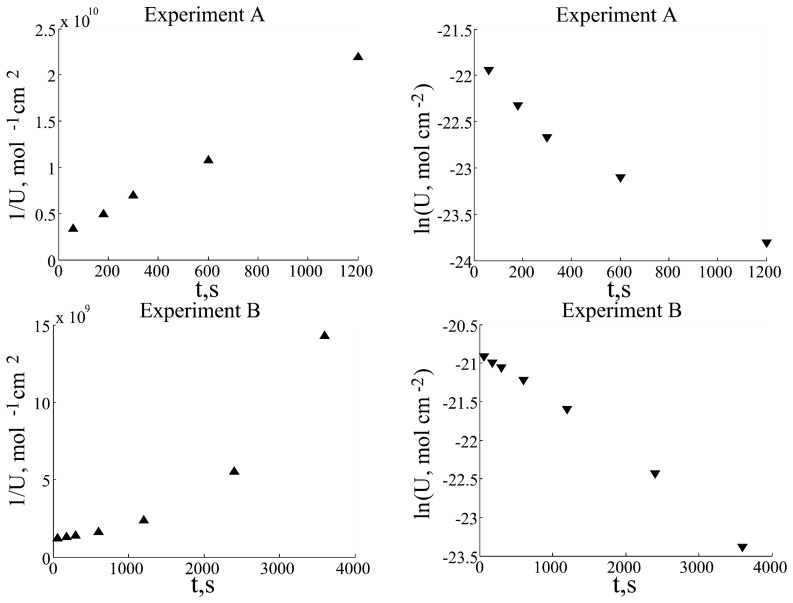
Experiment A and B data analysis: ▲- 1/U dependency on time; ▼- ln(U) dependency on time.

This model allowed good fitting only for a part of experimental data available (data not shown), which had strongly expressed exponential character of substrate concentration decrease (Fig. 2, experiment B; for the characteristics of different experiments, see parameters in the table).

However, another part of experimental data exhibited *U* decrease asymptotically proportional to t^-1^ (Fig 2, experiment A). Thus, the model of Eq. (3) was modified by adding a non-linear term of substrate wash off from the electrode surface, which allowed much better fitting results. Here, it should be noted that in work [5] the wash off effect of substrate has been observed experimentally in the solutions without added enzyme (see Fig. 1 in paper [5]). Therefore, we have reasonable grounds to believe that this process also occurs in the solutions containing enzyme.

Thus, slightly modified system of non-linear differential equations can be written by Eq. (4):

(4) $$\{\begin{array}{l} \frac{dE^{*}}{dt}=k_{-1}\;E^{*}U+k_{2}\;E^{*}U-k_{1}\;E^{*}\times U+k_{p}\;E-k_{D}\;E^{*}\\ \frac{dE^{*}U}{dt}=k_{1}\;E^{*}\times U-k_{-1}\;E^{*}U-k_{2}\;E^{*}U\\ \frac{dU}{dt}=k_{-1}\;E^{*}U-k_{1}\;E^{*}\times U-k_{u}\;{\left(\frac{U}{U_{0}}\right)}^{2}\\ \frac{dP}{dt}=k_{2}\;E^{*}U\\ \frac{dE}{dt}=\left(\frac{S}{V}\right)k_{D}\;E^{*}-\left(\frac{S}{V}\right)k_{p}\;E \end{array},$$

where definitions are the same as for Eq. (3), and *k*_u_ is the substrate wash off rate constant and *U*_0_ is the initial substrate concentration on the electrode surface.

Non-linear wash off term is quite unusual, but it could be explained in a simplified way as complex outcome of two different linear wash off rates: one for the electrode surface/substrate boundary (stronger bond, lower wash off rate) and second (weaker attraction, much higher wash off rate) for, say, substrate/substrate boundary. It is possible that during the process of modified electrode preparation substrate forms only very few substrate/substrate boundaries (pseudo-multilayer interfacial structure). Thus, initially wash off rate could be seen as linearly (in respect to the substrate concentration) dropping from high value for the substrate/substrate boundary, down to low value for the electrode/substrate boundary, and the whole process then becomes second order with respect to the substrate concentration. By way of illustration, let's assume that the wash-off rate constant *(k)* changes linearly with relative substrate concentration: *k*=*a*·*U*/*U_0_*+*b*, where *a* and *b* are the constants, so non-linearity could be introduced by substituting the wash-off rate constant in standard linear wash-off model: *dU*/*dt* = -*kU*.

## 3. Computer simulation setup and results

The series of computational simulations were performed to investigate how electrode readings would differ if this amperometric biosensor worked under presented model and how they would match experimental data (experimental results were obtained as described in [5], converting the integrated electrode peak current of the FPONDS-modified electrode to the surfaces concentration of ferrocene functional groups). The following values were used in our calculations: *V* = 4 cm^3^, *k*_2_ = 75 s^-1^, *k*_-1_ = 10 s^-1^, *k_p_* = 100 cm s^-1^, *k_D_* = 0.025 s^-1^, *S_A_* = 5.07×10^-2^ cm^2^, *S_B_* = 5.19×10^-2^ cm^2^, *S_C_* = 5.23×10^-2^ cm, *S_D_*=5.23×10^-2^ cm^2^. The values of four kinetic constants selected as a starting point for modeling were the same as in paper [6]. Besides, the following initial conditions were applied: *E(0)* = *E_0_*, E*(0) = E*S(0) = *P*(0) = 0, *U*(0) = *U*_0_. The values of initial *E_0_* and *U_0_* concentrations varied from experiment to experiment, *k_1_* and *k_u_* were subject of change for achieving better fitting (weighted least squares method was used) between experimental and simulation data. Non-linear ordinary equation system (4) was solved using Matlab (Matlab Release 14, The MathWorks Inc., Natick, USA) ODE solver for stiff problems. Solution time interval was 0..6000 seconds. The initial concentrations and best-fitted constants are presented in the Table 1.

**Figure 3.**
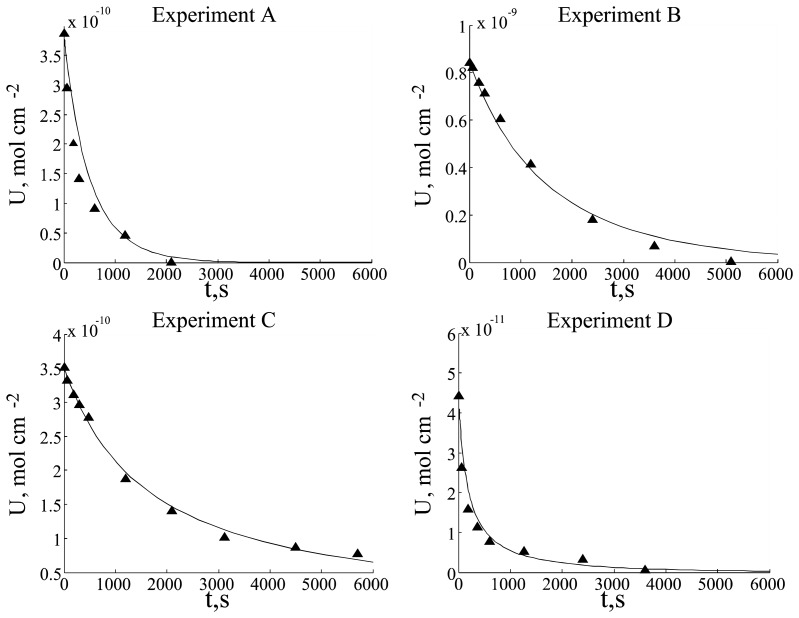
U dependency on time: solid line - simulation results, points – experimental data.

Experimental data and simulation results are presented in Fig. 3.

Experimental data were analyzed as logarithmic and *t*^-1^ graphs. These graphs reveal that experiments A, C and D strongly exhibit inverse dependence on time, whereas the data of experiment B has more exponential character. Such different graph characters could be explained as two term competition in *dU*/*dt* differential equation: first and second order (in respect to the substrate concentration) terms. First order term predominated over the second order term in experiment B, but second order term predominated over the first order term in experiments A, C and D. These observations enabled us to improve the model and to get better fitting between simulation and experimental data.

Finally, it is worth noting that the values of kinetic constant *k*_1_ obtained in this work are lower by ca. three orders of magnitude compared to the value reported in our earlier study [6]. Most likely, the difference is determined by different chemical nature of substrate head-groups in work [6] (dicyanohydroquinone-based group) and the present study (ferrocene-based group), since *k*_1_ reflects molecular event of substrate binding in the enzyme active center.

## Conclusions

The results of the foregoing computational experiments enable us to make the following conclusions:

1. The proposed reaction kinetic model of response of the FPONDS-based electrode, used for the electrochemical determination of *Thermomyces lanuginosus* lipase activity, allows to achieve a good fit between experimental data and simulation results.
2. According to the results of our study, experimental data exhibit two distinct types of substrate (FPONDS) concentration decay: one exponential (in respect to time) and the other of t^-1^-type. This indicates that, in the *dU*/*dt* differential equation in system Eq (4), first and second order (in respect to the substrate concentration) terms are competing and should be taken into account in numeric modeling.
3. Numeric simulations have revealed that a good fitting might be obtained only taking into account non-linear substrate wash off process, which could be explained in a simplified way as a complex outcome of two different linear wash off rates: one for the electrode surface/substrate boundary (stronger bond, lower wash off rate) and the other (weaker attraction, much higher wash off rate) for the substrate/substrate layer boundary. In this model of interface, it is assumed that substrate forms only very few substrate/substrate boundaries (pseudo-multilayer interfacial structure), thus wash off rate could be seen as linearly (with respect to the substrate concentration) dropping from high value for substrate/substrate boundary, down to low value for electrode surface/substrate boundary, therefore the whole process then becomes second order with respect to the substrate concentration.

## Figures and Tables

**Table 1.** Initial concentrations and best-fitted constants.

**Exp.**	***U****_0_***×10, mol cm^-2^**	***E****_0_***×10^12^, mol cm^-3^**	***k*_1_×10^-6^, mol cm^-2^ s^-1^**	***k*_u_×10^13^, mol cm^-2^ s^-1^**
A	3.88	58.0	0.41	2.26
B	8.43	5.80	1.20	2.13
C	3.51	0.58	1.17	2.06
D	0.44	8.30	0.75	2.34

## References

[1] Schmid R.D., Verger R. (1998). Lipases: interfacial enzymes with attractive applications. Angew. Chem. Int. Ed..

[2] Bornscheuerand U.T., Kazlauskas R.J. (1999). Hydrolases in Organic Synthesis Regio- and Stereoselective Biotransformations.

[3] Houde A., Kademi A., Leblanc D. (2004). Lipases and their industrial applications. Appl. Biochem. Biotechnol..

[4] Beisson F., Tiss A., Riviere C., Verger R. (2000). Methods for lipase detection and assay: a critical review. Eur. J. Lipid Sci. Technol..

[5] Valincius G., Ignatjev I., Niaura G., Kažem÷kait÷ M., Talakait÷ Z., Razumas V., Svendsen A. (2005). Electrochemical method for the dection of lipase activity. Anal. Chem..

[6] Puida M., Ivanauskas F., Ignatjev I., Valinčius G., Razumas V. (2007). Computational modeling of the amperometric bioanalytical system for lipase activity assay: a time-dependent response. Nonlinear Analysis: Modelling and Control.

[7] Verger R., Mieras M. C. E., De Haas G. H. (1972). Action of phospholipase A at interfaces. J. Biol. Chem..

